# Clinical, genetic profile and therapy evaluation of 11 Chinese pediatric patients with Fanconi-Bickel syndrome

**DOI:** 10.1186/s13023-024-03070-8

**Published:** 2024-02-16

**Authors:** Taozi Du, Yu Xia, Chengkai Sun, Zhuwen Gong, Lili Liang, Zizhen Gong, Ruifang Wang, Deyun Lu, Kaichuang Zhang, Yi Yang, Yuning Sun, Manqing Sun, Yu Sun, Bing Xiao, Wenjuan Qiu

**Affiliations:** 1 Department of Pediatric Endocrinology and Genetic Metabolism, Xinhua Hospital, Shanghai Institute of Pediatric Research, School of Medicine, Shanghai Jiao Tong University, 1665 Kongjiang Road, 200092 Shanghai, China; 2 Department of Clinical Genetics Centre, Xinhua Hospital, School of Medicine, Shanghai Jiao Tong University, 1665 Kongjiang Road, 200092 Shanghai, China

**Keywords:** Fanconi-Bickel syndrome, *SLC2A2*, Glucose transporter protein 2, Variant, Regions of homozygosity

## Abstract

**Background:**

Fanconi-Bickel syndrome (FBS) is a rare autosomal recessive disorder characterized by impaired glucose and galactose utilization as well as proximal renal tubular dysfunction.

**Methods:**

Clinical, biochemical, genetic, treatment, and follow-up data for 11 pediatric patients with FBS were retrospectively analysed.

**Results:**

Hepatomegaly (10/11), short stature (10/11) and hypophosphataemic rickets (7/11) were the most common initial symptoms. At diagnosis, all patients had decreased fasting blood glucose (FBG), plasma bicarbonate (HCO_3_^−^) and serum phosphorus, as well as elevated liver transaminases, alkaline phosphatase (AKP) and proximal renal tubular dysfunction. Two infant patients were misdiagnosed with transient neonatal diabetes mellitus. After therapy with uncooked cornstarch and conventional rickets treatment, remission of hepatomegaly was observed in all patients, with significant improvements in pre-prandial blood glucose, liver transaminases, triglyceride, plasma HCO_3_^−^ and AKP (*p* < 0.05). At the last follow-up, 5/7 patients with elevated AKP had nephrocalcinosis. The mean height standard deviation score (Ht SDS) of eight patients with regular treatment increased from − 4.1 to -3.5 (*p* = 0.02). Recombinant human growth hormone (rhGH) was administered to 4/9 patients, but their Ht SDS did not improve significantly (*p* = 0.13). Fourteen variants of the *SLC2A2* gene were identified, with six being novel, among which one was recurrent: c.1217T > G (p.L406R) (allele frequency: 4/22, 18%). Patients with biallelic missense variants showed milder metabolic acidosis than those with null variants. Two of five patients from nonconsanguineous families with rare homozygous variations showed 5.3 Mb and 36.6 Mb of homozygosity surrounding the variants, respectively; a region of homozygosity (ROH) involving the entire chromosome 3 covering the *SLC2A2* gene, suggesting uniparental disomy 3, was detected in one patient.

**Conclusions:**

Early diagnosis of FBS is difficult due to the heterogeneity of initial symptoms. Although short stature is a major issue of treatment for FBS, rhGH is not recommended in FBS patients who have normal GH stimulation tests. Patients with biallelic null variants may require alkali supplementation since urine bicarbonate loss is genetically related. ROH is a mechanism for rare homozygous variants of FBS in nonconsanguineous families.

**Supplementary Information:**

The online version contains supplementary material available at 10.1186/s13023-024-03070-8.

## Introduction

Fanconi-Bickel syndrome (FBS, OMIM 227,810) is a rare autosomal recessive disorder of glucose transport [1]. The deficiency of glucose transporter 2 (GLUT2), a monosaccharide carrier that is responsible for the transport of both glucose and galactose, results in hepatomegaly, fasting hypoglycaemia, post-prandial hyperglycaemia, transient neonatal diabetes mellitus (TNDM), generalized proximal tubular dysfunction, hypophosphataemic rickets and growth retardation. The incidence of FBS is still unclear, with approximately 220 reported cases worldwide [2–13].

FBS is caused by variants in the *SLC2A2* gene, which result in dysfunction of GLUT2 [14, 15]. The human *SLC2A2* gene, located at 3q26.1-q26.3, consists of 11 exons [16]. GLUT2 is expressed in the liver, intestine, kidney, the central nervous system and pancreas. It plays a crucial role in maintaining glycaemic stability by regulating glucose influx and efflux in hepatocytes, glucose absorption in intestinal epithelial cells, glucose reabsorption in renal proximal tubule cells and glucose sensing in the central nervous system [11, 17]. Although GLUT2 is not the predominant glucose transporter in human pancreatic β-cells, it is temporarily necessary for regulating insulin secretion during the early months of life [11]. Current treatment mainly focuses on stabilizing glucose levels and compensating for the loss of different solutes in kidneys. This includes replacing vitamin D, phosphate and/or sodium bicarbonate, restricting galactose intake, following a diet similar to that of individuals with diabetes mellitus and administering uncooked cornstarch (UCS). A recent study found that continuous nocturnal gastric drip feeding could effectively restore growth failure in some cases [12].

FBS is very rare and often misdiagnosed as other conditions, such as neonatal diabetes mellitus (NDM) or rickets. Therefore, to deepen our comprehension of this disease, we retrospectively evaluated the clinical, biochemical, and genetic characteristics, as well as treatment, of 11 patients diagnosed with FBS in our centre. In addition, we investigated the mechanism for rare homozygous variants of FBS in nonconsanguineous families.

## Patients and methods

### Patient recruitment

Patients who met the following criteria were enrolled: (i) harbouring biallelic *SLC2A2* variants classified as pathogenic (P) or likely pathogenic (LP); and (ii) FBS phenotypes (hepatomegaly, dysglycaemia, proximal renal tubular nephropathy, hypophosphataemic rickets and short stature). This study received approval from the ethics committee of Xinhua Hospital, Shanghai Jiao Tong University School of Medicine (XHEC-D-2022-271). Written informed consent was obtained from the patients or their legal guardians.

### Study design

A total of eleven eligible subjects were included in this study. The medical data obtained from the patients’ files were as follows: (i) demographic information; (ii) age at onset, initial visit, diagnosis and the last follow-up; (iii) birth weight; (iv) height; (v) clinical signs/symptoms and laboratory findings; (vi) *SLC2A2* genetic results; and (vii) the treatment administered and its efficacy during follow-up.

Hepatomegaly was evaluated by physical examination and abdominal ultrasound. The presence of hypophosphataemic rickets was evaluated by radiography of the knees, wrists and/or ankles. The biochemical parameters measured included fasting blood glucose (FBG), pre-prandial blood glucose (PBG); serum alanine aminotransferase (ALT), aspartate transaminase (AST), γ-glutamyl transferase (GGT), alkaline phosphatase (AKP), phosphorus, calcium, total cholesterol (TC), triglyceride (TG), uric acid (UA), insulin-like growth factor-1 (IGF-1); plasma pH, bicarbonate (HCO_3_^−^), base excess (BE), lactate; glycosuria, proteinuria and ketonuria. Reference ranges for the laboratory parameters are listed in Table S1. Values of serum AKP, phosphorus, UA, IGF-1 and height were converted to the standard deviation score (SDS) due to different reference values based on age and sex [18–23].

The follow-up period for this study was from January 2007 to July 2023. The treatment includes replacement of phosphate [20∼60 mg/kg/d (0.7∼2.0 mmol/kg/d)] and vitamin D (Calcitriol, 20∼30 ng/kg/d), restriction of galactose, administration of UCS (1.6∼2.5 g/kg for 4∼6 times daily) and a diabetes mellitus-like diet (presented in frequent small meals with adequate caloric intake) [24–26]. Alkali (sodium bicarbonate/sodium citrate, 5∼20 mEq/kg/d) was provided to patients who had consistent metabolic acidosis [27]. The recommended diet contained 60∼70% calories from carbohydrates, 10∼15% from protein, and the remaining from fat [28]. Recombinant human growth hormone (rhGH) was administered to four patients with significantly short stature. The dose was 0.1∼0.2 IU/kg/d, and dosages were adjusted according to growth velocity (GV) and IGF-1 level [29].

### Molecular analysis

Genomic DNA was extracted from the peripheral blood of the patients and their parents using the QIAamp DNA blood mini kit (QIAGEN, Valencia, CA, USA). Seven patients underwent PCR and Sanger sequencing, and four patients were tested by exome sequencing (ES). The genetic test procedures were in Additional file 2.

### Statistical analysis

Statistical analysis was executed by GraphPad Prism 9.0 (GraphPad Software Inc. San Diego, CA, USA; www.graphpad.com). Normally distributed, nonnormally distributed, and categorical variables are presented as means ± SDs, medians (interquartile ranges), and frequencies, respectively. Age at onset, diagnosis and the last follow-up are presented as means (range: min∼max). The paired Student’s t test or Wilcoxon test was used to compare differences between two paired groups. The unpaired Student’s t test or Mann‒Whitney U test was used to compare differences between two independent groups. Fisher’s exact test was used for two categorical variables. The level of significance was set at 0.05 (two-tailed).

## Results

### Demographic features

Eleven patients (7 males and 4 females) from ten families were diagnosed with FBS. Except for one patient (P7), the remaining ten patients were born to nonconsanguineous marriage partners. The mean age at onset and diagnosis was 1.3 years (range: 3 days∼2.3 years) and 2.2 (range: 1.1∼3.4) years, respectively (Table 1).

### Initial clinical and biochemical features

The initial detailed clinical and biochemical information is shown in Table 1 and Table S2. Six patients were referred to our centre for abdominal distension and short stature (P4, P7, P8, P9i, P9ii and P10), three for respiratory infections (P3, P5 and P6), one for malnutrition (P2) and the remaining one for hyperglycaemia after birth (P1) at their initial visit. Hepatomegaly was observed in 10/11 patients, except for P1, who visited in the neonatal period. Decreased FBG (2.0 ± 0.5 mmol/L) was detected in 9/11 patients. Elevated ALT [128.0 (65.0, 248.3) U/L] was observed in 6/10 patients, elevated AST [121.0 (67.5, 197.0) U/L] in 9/10 and elevated GGT (134.1 ± 98.4 U/L) in 8/8. TC (P4: 7.30 mmol/L; P9i: 7.16 mmol/L) was increased in 2/7 patients and TG (4.0 ± 1.2 mmol/L) in 6/7. The mean lactate level was 2.2 ± 0.8 mmol/L (*n* = 6). The median UA SDS was − 3.5 (-3.5, -3.1) (*n* = 7).

Renal proximal tubular disorders were observed in all 11 patients. Glycosuria was detected in 11/11 patients, proteinuria in 7/11 and ketonuria in 6/11. Metabolic acidosis was confirmed in all patients with a markedly decreased plasma HCO_3_^−^ level (15.0 ± 3.2 mmol/L). The mean pH and BE were 7.35 ± 0.04 (*n* = 11) and − 9.7 ± 2.5 mmol/L (*n* = 11), respectively. Except for P1 without records, ten patients showed elevated AKP [7.7 (5.5, 15.9) SDS], decreased serum phosphorus (-6.2 ± 1.1 SDS) and normal serum calcium (2.39 ± 0.18 mmol/L). Seven patients received bone X-rays, which showed rickets imaging changes, such as cupped and flared metaphyses along with widened and irregular growth plates of the long bones (*n* = 7), varus deformity (P4) and valgus deformity (P10). Low birth weight (< 2500 g) was observed in 2/11 patients (P1 and P9ii). Short stature [height SDS (Ht SDS): -3.4 ± 0.9] was observed in 10/11 patients at the first visit, except for P1.

Two patients (P1 and P2) were initially misdiagnosed with NDM due to the incidental detection of hyperglycaemia, rather than the presence of classical symptoms of diabetes mellitus at the ages of 3 days and 4 months, respectively. P1 was diagnosed with NDM based on increased random blood glucose (11.8∼12.4 mmol/L) with decreased fasting C-peptide (0.33 nmol/L, reference range: 0.37∼1.47 nmol/L) and insulin (10.2 pmol/L, reference range: 13.0∼161.0 pmol/L) at three days after birth. Insulin antibodies were negative. Her HbA1c level (6.4%, reference range: 4∼6%) did not fully meet the diagnostic criteria for diabetes mellitus (HbA1c < 6.5%). P1 was treated with insulin (0.15 U/kg/d) at the age of one month. Due to hypoglycaemia (2.0∼3.0 mmol/L) and frequent drowsiness, insulin was changed to glibenclamide (0.06 mg/kg/d) at the age of three months, which was discontinued after the genetic diagnosis at the age of 20 months. P2 was observed to have hyperglycaemia and glycosuria when he was four months old. In the fasting state, elevated blood glucose (9.5 mmol/L), decreased C-peptide (0.08 nmol/L) and low insulin (< 1.4 pmol/L) were detected. The 2-hour blood glucose level based on the oral glucose tolerance test was 15.0 mmol/L, which met the diagnostic criteria for diabetes mellitus. However, the level of HbA1c was 6.3%, and the insulin antibody test was negative. Then, he was diagnosed with NDM and initiated insulin therapy (1.2 U/kg/d), which was discontinued due to frequent episodes of hypoglycaemia (approximately 3 mmol/L) at the age of 6 months.

### Clinical and biochemical features at diagnosis

All patients were diagnosed at the mean age of 2.2 (range: 1.1∼3.4) years through gene sequencing. The median gap between the initial visit and diagnosis was 2.0 (range: 0.0∼15.0) months. All patients showed decreased FBG, elevated ALT, elevated GGT, decreased UA, elevated AKP, decreased serum phosphorus, normal serum calcium, decreased plasma HCO_3_^−^, decreased BE and glycosuria (Table S3). Hepatomegaly and short stature (Ht SDS: -4.1 ± 1.2) were also observed in all patients. Rickets imaging changes were observed in all patients, and skeletal deformities were found in 5/11.

### Clinical and biochemical features after treatment (*n* = 9)

P2 and P3 were lost to follow-up. The remaining nine patients were administered UCS, conventional rickets treatment (oral active vitamin D and phosphate) and sodium bicarbonate after diagnosis. The average treatment duration was 7.0 (range: 1.8∼15.5) years. Detailed clinical and biochemical information is provided in Table 1 and Table S4. At the last follow-up, remission of hepatomegaly was observed in all nine patients. Levels of PBG, ALT, AST and TG returned to almost normal (all *p* < 0.05), whereas levels of GGT, TC, lactate and UA were not different from pre-treatment (all *p* > 0.05).

There was no significant difference in the prevalence of glycosuria, proteinuria and ketonuria before and after treatment (all *p* > 0.05). Metabolic acidosis moderately improved after treatment, as the median plasma HCO_3_^−^ level increased significantly from 14.6 to 21.4 mmol/L. Nevertheless, pH and BE levels did not differ significantly from pre-treatment (both *p* > 0.05), and six patients (P1, P4, P8, P9i, P9ii and P10) still showed acidosis. Median AKP SDS decreased significantly from 9.2 to 3.6, while seven patients (except P6 and P9ii) still exhibited elevated AKP. Serum phosphorus and calcium levels were not significantly different (both *p* > 0.05). Except for P8, eight patients presented with skeletal deformities. Five patients (P5, P6, P9i, P9ii and P10) had nephrocalcinosis at the last follow-up, three of whom still had elevated AKP levels.

**Fig. 1:**
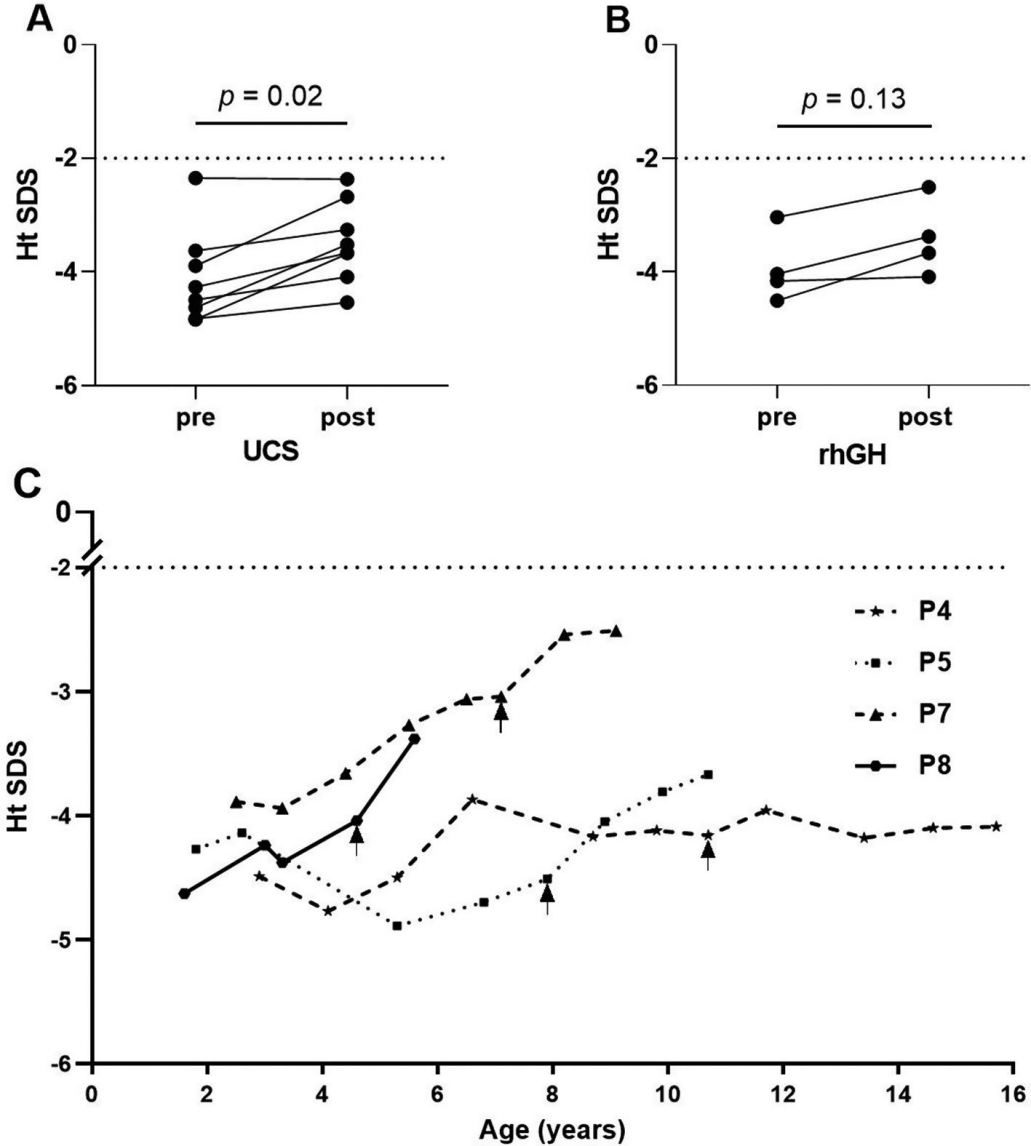
Height SDS of our FBS patients. (**A**) The Height SDS of eight patients before and after UCS therapy except P1. (**B**) The Height SDS of four patients before and after rhGH therapy. (**C**) Changes in Height SDS of four patients receiving rhGH therapy since the initiation of UCS therapy. Arrows mark when rhGH treatment started. Ht SDS: height standard deviation score; UCS: uncooked cornstarch; rhGH: recombinant human growth hormone

The mean age of these nine patients at the last follow-up was 8.9 (range: 3.1∼16.6) years. P1 and P4 entered adolescence at the age of 14 years and 13.3 years, respectively, indicating that there was a delay of puberty. The final adult height of P1 (110.1 cm, Ht SDS: -9.14) deviated significantly from the normal range, potentially due to her lack of consistent adherence to UCS and rickets treatment for a long term. The Ht SDS of the remaining eight patients who continuously adhered to the UCS and rickets treatment for 4.5 ± 2.5 years increased significantly from − 4.1 ± 0.8 to -3.5 ± 0.7 (*p* = 0.02) (Fig. 1A). Four patients (P4, P5, P7, and P8) exhibited a pronounced deficiency of Ht SDS [-4.1 (-4.4, -3.3)]. Additionally, these patients displayed a reduction in median IGF-1 levels [-2.1 (-2.6, -1.3) SDS]. A growth hormone (GH) provocative test was conducted, which yielded results indicating adequate levels of GH. Despite receiving rhGH treatment for an average duration of 2.8 (range: 1.0∼5.0) years, the four patients did not exhibit a significant improvement in their Ht SDS (from − 4.1 to -3.6, *p* = 0.13) (Fig. 1B). This suggests that rhGH therapy has limited efficacy in promoting height gain in individuals with FBS. The changes in Ht SDS and GV of these four patients who underwent rhGH treatment are shown in Fig. 1C. One patient (P8) had rhGH treatment for a duration of one year, while the remaining three (P4, P5 and P7) for a median duration of 2.9 (range: 2.1∼5.0) years. Following the initial year of rhGH therapy, the median GV of the four patients exhibited an increase from 5.5 to 7.3 cm/year (*p* = 0.13). As the duration of treatment increased, there was a drop in the median GV to 4.5 (2.6, 4.8) cm/year, which was lower than the levels observed prior to GH treatment, providing more evidence that rhGH had limited effectiveness.

**Table 1:** Demographic and clinical information of 11 patients with FBS

**Demographic information**		
Male/female (n)	7/4	
Age at onset (d/y) (*n* = 11)	1.3 y (3 d∼2.3 y)	
Age at diagnosis (y) (*n* = 11)	2.2 (1.1∼3.4)	
Follow-up duration (y) (*n* = 9)	6.7 (1.4∼15.2)	
**Clinical information**		
**Initial symptoms (n)**		
Hepatomegaly	10/11	
Short stature	10/11	
Hypophosphataemic rickets	7/11	
**Biochemical parameters (*n*** **= 9)**	**Pre-treatment**	**Post-treatment**
Glucose (mmol/L) *	2.0 ± 0.7	4.5 ± 0.7
ALT (U/L) *	98.3 ± 56.8	30.4 ± 8.7
AST (U/L) *	121.0 (66.0, 276.5)	34.0 (30.0, 86.0)
GGT (U/L)	55.0 (39.5, 145.5)	43.0 (27.5, 168.5)
TC (mmol/L)	5.1 ± 1.2	5.2 ± 1.0
TG (mmol/L) *	3.6 ± 1.8	1.4 ± 0.4
UA SDS	-3.1 ± 0.4	-3.2 ± 0.5
Lactate (mmol/L)	1.8 ± 0.7	1.5 ± 0.6
pH	7.34 ± 0.06	7.32 ± 0.06
HCO_3_^−^ (mmol/L) *	14.6 (12.4, 17.0)	21.4 (14.7, 23.2)
BE	-10.3 ± 3.0	-7.4 ± 5.6
AKP SDS *	9.2 (6.4, 10.5)	3.6 (1.6, 6.3)
Phosphorus SDS	-6.4 ± 1.7	-5.6 ± 1.0
Calcium (mmol/L)	2.26 (2.25, 2.51)	2.40 (2.29, 2.57)

Data are shown as n, means (ranges), medians (interquartile ranges) or means ± standard deviations. d: day; m: month; y: year. *: showing significant difference. ALT: alanine aminotransferase; AST: aspartate transaminase; GGT: γ-glutamyl transferase; TC: total cholesterol; TG: triglycerides; UA SDS: uric acid standard deviation score; HCO_3_^−^: bicarbonate; BE: base excess; AKP SDS: alkaline phosphatase SDS

### *SLC2A2* variation spectrum of this cohort and genotype-phenotype analysis

**Fig. 2:**
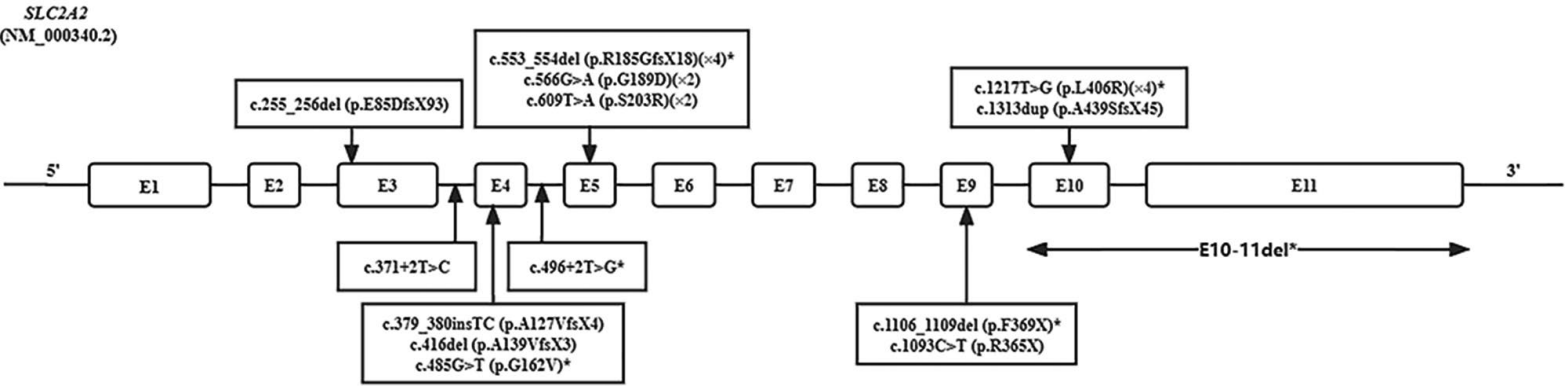
Variants of *SLC2A2* gene identified in 11 patients with FBS. The *SLC2A2* variants were depicted as nucleotide alterations and amino acid alterations per exon/intron. The white boxes indicated exons. Novel variants were marked with an asterisk. The suffixes indicated the number of mutant alleles

Based on the results of Sanger sequencing and ES, 11 patients were found to harbour biallelic P/LP variants in the *SLC2A2* gene (Fig. 2 and Table S5). Fourteen variants were identified, including missense (*n* = 4), nonsense (*n* = 2), frameshift (*n* = 5), splicing site variants (*n* = 2) and gross deletion (*n* = 1). Six variants are novel, among which one was recurrent: c.1217T > G (p.L406R) (allele frequency: 4/22, 18%) in two unrelated homozygous patients.

Nonsense, frameshift, splicing site variants and gross deletion could be classified as null variants. An attempt was made to investigate whether six patients (P2, P4, P8, P9i, P9ii and P10) with null variants in biallelic alleles had earlier symptoms or more severe biochemical changes than the four patients (P1, P3, P6 and P7) with missense variants in biallelic alleles. Except for plasma HCO_3_^−^ and BE, other biochemical parameters and signs/symptoms were not different between these two groups. Levels of plasma HCO_3_^−^ and BE in these four patients with missense variants at diagnosis were 17.8 (16.1, 19.7) mmol/L and − 7.4 (-8.3, -6.3) mmol/L, respectively, which were significantly higher than those [plasma HCO_3_^−^: 12.9 (11.9, 13.7) mmol/L, BE: -12.3 (-13.4, -11.3) mmol/L] of the six patients with biallelic null variants (both *p* = 0.01). Therefore, we speculate that patients with missense variants have milder acidosis than those with null variants. Compared with missense variants, null variants are likely to cause more severe urinary bicarbonate loss, indicating a relationship between genotype and phenotype (Table 2).

**Table 2:** Patients with different types of *SLC2A2* variants and the levels of plasma HCO_3_^−^ and BE

Variant type in both alleles	Patient ID	Allele 1 (paternal)		Allele 2 (maternal)		HCO_3_^−^(mmol/L)	BE (mmol/L)
		Nucleotide alteration	Amino acid alteration	Nucleotide alteration	Amino acid alteration		
Missense	P1	c.609T > A	p.S203R	c.609T > A	p.S203R	16.2	-8.3
	P3	c.1217T > G*	p.L406R	c.1217T > G*	p.L406R	19.8	-6.4
	P6	c.1217T > G	p.L406R	c.1217T > G	p.L406R	16.1	-8.3
	P7	c.566G > A	p.G189D	c.566G > A	p.G189D	19.4	-6.2
Nonsense, frameshift, splicing site or gross deletion	P2	c.1313dup	p.A439SfsX45	c.379_380insTC	p.A127VfsX4	13.1	-11.9
	P4	c.255_256del	p.E85DfsX93	c.496 + 2T > G	/	12.6	-11.4
	P8	E10-11del	/	c.1106_1109del	p.F369X	13.4	-10.8
	P9i	c.553_554del	p.R185GfsX18	c.553_554del	p.R185GfsX18	12.1	-12.9
	P9ii	c.553_554del	p.R185GfsX18	c.553_554del	p.R185GfsX18	14.6	-12.6
	P10	c.1093 C > T	p.R365X	c.371 + 2T > C	/	11.3	-14.9

HCO_3_^−^: bicarbonate; BE: base excess

/: unknown amino acid alteration

*variant with unknown origin

In our study, 6/11 patients were homozygous for rare disease-causing variations, with only one case (P7) from a consanguineous family. The remaining five patients (P1, P3, P6, P9i and P9ii) were from nonconsanguineous families. To determine the mechanism of homozygous rare variants in these families, the ES data of P9i was re-analysed, and the ROH around the homozygous causal variant was studied. P9i carried a stretch of 36.6 Mb homozygosity (chr3: 142,222,074–178,785,653, hg19) around the homogenous variant. Her ROH was 1.6% of the entire genome, which is similar to the inbreeding coefficient of marriage between second cousins. Then, newly obtained ES data for P1, P3 and P6 were analysed for ROH. P1 was found to carry 5.3 Mb of homozygosity (chr3: 168,269,512 − 173,525,731, hg19) surrounding the homogenous variant. Her ROH was 0.4% of the whole genome, equivalent to the inbreeding coefficient of a third-cousin marriage. P3 was discovered to carry an ROH area of whole chromosome 3 involving the *SLC2A2* gene, implying uniparental disomy (UPD) 3. However, we were unable to determine whether the two copies of chromosome 3 originated from the same parent. ROH surrounding the homozygous variant was not found in P6.

## Discussion

Glycogen storage diseases (GSDs) are a group of congenital abnormalities affecting glycogen metabolism and are characterized by the proteins and tissues affected [30]. FBS is an extremely rare type of hepatic GSDs, which is found in 0.92% (11/1198) of our centre’s patients with hepatic GSDs, underlining its rarity in the Chinese population. The heterogeneity of initial symptoms in FBS patients makes early diagnosis challenging. Current treatment is effective in maintaining blood glucose and improving liver function, but there are still limitations in terms of rickets, acidosis and secondary short stature.

TNDM can be observed in FBS patients who develop hyperglycaemia within the first six months of life, and spontaneously resolve after a few months [31]. Hyperglycaemia may be related to impaired glucose-stimulated insulin secretion by pancreatic β-cells, leading to reduced uptake of glucose [32]. Its self-remission may be attributed to GLUT1 and GLUT3, which replace GLUT2 as the major glucose transporter in human β-cells after the neonatal and developmental periods [11, 33]. TNDM was diagnosed in 2/11 cases (P1 and P2) in our cohort based on incidentally detected hyperglycaemia. However, their HbA1c levels (P1: 6.4%; P2: 6.3%) were only marginally elevated, and classical symptoms of diabetes mellitus were not observed, which was consistent with a previous study [34]. These two patients experienced frequent hypoglycaemia after insulin therapy. Therefore, mildly elevated HbA1c levels, absence of classical symptoms and frequent insulin-induced hypoglycaemia may be clinical features of TNDM in FBS patients. Insulin therapy may cause frequent episodes of hypoglycaemia in FBS patients [34]. As a result, it is critical to detect TNDM caused by FBS in a timely manner. In other words, when TNDM patients present with these clinical signs, FBS should be considered, especially if they are accompanied by characteristic FBS markers such as liver function abnormalities, high AKP, and decreased serum phosphorus.

All of our patients were treated with conventional rickets therapy [24]. At the last follow-up, elevated AKP was still observed in 7/9 patients, suggesting that the current therapy regimen can only partially correct rickets in FBS patients. Furthermore, nephrocalcinosis was identified in 5/9 individuals, with three patients still having elevated AKP levels. In FBS, storage of glycogen leads to generalized proximal tubular dysfunction, which contributes to the presentation of hypercalciuria, hyperphosphaturia and calcium phosphate deposition in the kidneys [35, 36]. Conventional rickets treatment may also increase urinary excretion of calcium and phosphorus, further raising the risk of nephrocalcinosis. Thus, it is difficult to restore the AKP level to normal while also preventing nephrocalcinosis with existing treatment. A recent study shown that sodium glucose cotransporter 2 (SGLT2) inhibitor could increase the levels of serum potassium and phosphate concentrations in FBS, indicating that SGLT2 inhibitor have promise as a novel therapeutic option [37].

Metabolic acidosis is a prevalent characteristic of FBS caused by ketone accumulation and bicarbonate loss through the proximal renal tubules [27, 38]. The accumulation of ketone is a reflection of inadequate management or fasting intolerance in GSD. Ketone has a strong monitoring value for treatment and dietary treatment is the cornerstone to prevent hyperketosis [39, 40]. The metabolic acidosis observed in FBS primarily originated from the kidneys and was likely caused by a malfunction in the proximal tubule [38]. All patients in our cohort had varying degrees of metabolic acidosis during their initial visit, which was in line with previous studies [6, 13, 41]. There is no consensus on alkali therapy for FBS patients. After UCS and conventional rickets treatment, plasma HCO_3_^−^ levels in our three patients normalized, who harboured at least one missense variant. While, metabolic acidosis did not improve appreciably in the other six patients who received the same treatment and carried null variants in both alleles. This shows that the amount of urine bicarbonate loss may be genetically associated and that patients with biallelic null variants require additional alkali supplementation. The correlation between the severity of acidosis and the type of variant has not been reported in previous literature [10, 11, 42, 43]. Sharari et al. conducted an analysis on the severity of dysglycaemia in 144 reported patients [11]. The findings of their analysis yielded a negative conclusion, which aligns with the results of our analyses.

According to earlier studies, the Ht SDS of thirteen FBS patients who reached the final height ranged from − 9.5 to -1.6 [1, 3, 12, 44, 45], demonstrating that short stature is one of key problems in clinical management of FBS [6, 12, 13, 46]. Short stature can be attributed to reduced chondrogenesis at the growth plates [47, 48]. Growth plate chondrogenesis is influenced by several factors, such as intracellular regulatory mechanisms in the chondrocytes, cartilage extracellular matrix components, paracrine factors and endocrine factors [47]. Multiple causes, including hypoglycaemia, acidosis, and hypophosphataemia, may contribute to the short stature occurring in FBS. Hypoglycaemia and acidosis have an impact on the GH/IGF-1 axis, resulting in decreased cellular proliferation at the growth plate [49]. FBS patients exhibit lower IGF-1 levels [50], with − 2.1 ± 0.7 SDS in our patients (*n* = 5), which is likely attributable to the potential occurrence of hypoglycaemia. Furthermore, hypophosphataemia in FBS could affect mineralization at the growth plate [51]. The adult Ht SDS of X-linked hypophosphataemic rickets ranges between − 2 and − 1 [49, 52, 53], indicating that height is more severely affected in FBS. After 4.5 years of treatment, the Ht SDS of nine patients in our group increased by only 0.6, indicating that the effect of current treatment on height is modest and consistent with the previous research [54]. rhGH administration for 2.8 years in our four patients with extremely short stature also showed a limited benefit, with the median Ht SDS only increasing from − 4.1 to -3.7. Our results suggest that rhGH should not be indicated for FBS patients with normal GH stimulation tests.

To date, 109 different variants of *SLC2A2* have been reported in the HGMD database (http://www.hgmd.cf.ac.uk/ac/). In our study, we identified 14 *SLC2A2* variants and they are scattered over the whole coding sequence, without any obvious pattern. One recurrent variant, c.1217T > G (p.L406R) (allele frequency: 4/22, 18%), was identified in two unrelated homozygous cases in our study, which was also not reported previously. The hotspot/founder variants in India, Sudan and Turkey are c.952G > A (p.G318R) (4/10, 40%), c.157 C > T (p.R53*) (6/10, 60%), and c.482_483insC (p.G162Rfs*17) (6/8, 75%) [6, 13, 41], respectively, which have not been found in Chinese patients. According to the large frequency of consanguineous relatives, 74% of patients with genetic diagnosis carry homozygous variants [55]. However, of the six patients in our study who harboured homozygous variants, only one (P7) was from a consanguineous family. Long stretches of homozygosity around homozygous rare pathogenic variants have been observed in nonconsanguineous families with rare AR diseases [56]. In our study, two patients (P1 and P9i) from nonconsanguineous families were also found to have stretches of homozygosity (5.3 Mb and 36.6 Mb) around the homogenous variants, which is equivalent to the inbreeding coefficient of third and second cousins, respectively. These findings support that inbreeding over many generations does result in significant genome sharing and homozygosity for variations inherited from common ancestors [56]. Moreover, UPD is another type that might cause homogeneous rare variations. P3 showed an ROH region of whole chromosome 3, indicating the possible existence of UPD. It is crucial to determine the molecular basis of homozygous rare variants in nonconsanguineous families to provide genetic counselling and recurrent risk evaluation.

There are several limitations to our study. First, due to the low incidence, it was a retrospective study with a small sample size of patients, and data on some biochemical parameters was missing, which may result in bias. Second, since the follow-up duration was insufficient, only two of the eleven patients had reached adolescence, which limited analyses of delayed puberty in FBS. Additionally, only one of the patients reached adult height. Thus, we were unable to assess if the current treatment regimen can effectively improve the final height of FBS patients, which calls for large-scale and long-term studies.

## Conclusions

In summary, we report eleven patients with FBS, deepening understanding of the clinical manifestations, genetic spectrum and treatment outcomes of this disorder. The heterogeneity of initial symptoms in FBS patients makes early diagnosis challenging. Current treatment is effective in preventing hypoglycaemia and improving liver function, but treatment of rickets, acidosis, and short stature continues to be challenging. Growth hormone therapy appears not to enhance growth in FBS patients who have normal GH stimulation tests. Patients with null variants in biallelic alleles may exhibit more severe metabolic acidosis and require alkali supplementation. ROH is a mechanism for assessing rare homozygous variants of FBS in the absence of a consanguineous family history.

### Electronic supplementary material

Below is the link to the electronic supplementary material.

**Additional file 1. Table S1:** Age-and sex-matched laboratory reference ranges. **Table S2:** Clinical and biochemical information of 11 patients with FBS at initial visit. **Table S3:** Clinical and biochemical information of 11 patients with FBS at diagnosis. **Table S4:** Clinical and biochemical information of 11 patients with FBS at the last follow-up. **Table S5:***SLC2A2* variants in 11 patients with FBS.

**Additional file 2.** The detailed genetic test procedures.

## Data Availability

All data generated or analysed during this study are included in this published article and its supplementary information files.

## Abbreviations

FBS: Fanconi-Bickel syndrome
TNDM: Transient neonatal diabetes mellitus
GLUT2: Glucose transporter protein 2
UCS: uncooked cornstarch
NDM: Neonatal diabetes mellitus
FBG: Fasting blood glucose
PBG: Pre-prandial blood glucose
ALT: Alanine aminotransferase
AST: Aspartate transaminase
GGT: γ-glutamyl transferase
TC: Total cholesterol
TG: Triglyceride
UA: Uric acid
HCO_3_^-^: Bicarbonate
BE: Base excess
IGF-1: Insulin-like growth factor-1
SDS: Standard deviation score
rhGH: Recombinant human growth hormone
GV: Growth velocity
ES: Exome sequencing
ROH: Regions of homozygosity
Ht SDS: Height SDS
GH: Growth hormone
UPD: Uniparental disomy
