# Supplementary material for: Clinical, genetic profile and therapy evaluation of 11 Chinese pediatric patients with Fanconi-Bickel syndrome
Source: Orphanet J Rare Dis. 2024 Feb 16;19:75. doi: 10.1186/s13023-024-03070-8 (PMC10874070; doi:10.1186/s13023-024-03070-8)
Supplement: Supplementary file 1 — Additional file 1. Table S1: Age-and sex-matched laboratory reference ranges. Table S2: Clinical and biochemical information of 11 patients with FBS at initial visit. Table S3: Clinical and biochemical information of 11 patients with FBS at diagnosis. Table S4: Clinical and biochemical information of 11 patients with FBS at the last follow-up. Table S5:SLC2A2 variants in 11 patients with FBS. [file 13023_2024_3070_MOESM1_ESM.docx]

**SUPPLEMENTAL TABLES:**

**Clinical, genetic profile and therapy evaluation of 11 Chinese pediatric patients with Fanconi-Bickel syndrome**

**Taozi Du MB ^1,#^, Yu Xia MB ^1,#^, Chengkai Sun MB ^1,#^, Zhuwen Gong MD ^1^, Lili Liang MD, PhD ^1^, Zizhen Gong MD ^1^, Ruifang Wang MD, PhD ^1^, Deyun Lu MD ^1^, Kaichuang Zhang MD ^1^, Yi Yang MD ^1^, Yuning Sun MD, PhD ^1^, Manqing Sun MD, PhD ^1^, Yu Sun MD, PhD ^1,2^, Bing Xiao MD, PhD ^1,2,*^, Wenjuan Qiu MD, PhD ^1,*^**

**^1^ Department of Pediatric Endocrinology and Genetic Metabolism, Xinhua Hospital, Shanghai Institute of Pediatric Research, School of Medicine, Shanghai Jiao Tong University, 1665 Kongjiang Road, Shanghai 200092, China**

**^2^ Department of Clinical Genetics Center, Xinhua Hospital, School of Medicine, Shanghai Jiao Tong University, 1665 Kongjiang Road, Shanghai 200092, China**

**Table S1.** Age-and sex-matched laboratory reference ranges

| **Laboratory** | **Age** | **Normal range (male)** | **Normal range (female)** |
| --- | --- | --- | --- |
| **Blood glucose (mmol/L)** | All | 3.9~6.1 | 3.9~6.1 |
| **Alanine aminotransferase (U/L)** | 28 days to < 1 year | 8~71 | 8~71 |
|  | 1 to < 2 years | 8~42 | 8~42 |
|  | 2 to < 13 years | 7~30 | 7~30 |
|  | 13 to 18 years | 7~43 | 6~29 |
| **Aspartate transaminase (U/L)** | 28 days to < 1 year | 21~80 | 21~80 |
|  | 1 to < 2 years | 22~59 | 22~59 |
|  | 2 to < 13 years | 14~44 | 14~44 |
|  | 13 to 18 years | 12~37 | 10~31 |
| **γ-glutamyl transferase (U/L)** | 28 days to < 6 months | 9~150 | 9~150 |
|  | 6 months to < 1 year | 6~31 | 6~31 |
|  | 1 to < 13 years | 5~19 | 5~19 |
|  | 13 to 18 years | 8~40 | 6~26 |
| **Serum calcium(mmol/L)** | 28 days to 18 years | 2.1~2.8 | 2.1~2.8 |
| [**Total cholesterol**](javascript:;) **(mmol/L)** | All | 3.36~6.46 | 3.36~6.46 |
| **Triglyceride (mmol/L)** | All | 0.20~2.31 | 0.20~2.31 |
| **pH** | All | 7.35~7.45 | 7.35~7.45 |
| **Plasma bicarbonate (mmol/L)** | All | 22~27 | 22~27 |
| **Base excess (mmol/L)** | All | -3~3 | -3~3 |
| **Lactate (mmol/L)** | All | 0.7~2.1 | 0.7~2.1 |

**Table S2.** Clinical and biochemical information of 11 patients with FBS at initial visit

|  | **P1** | **P2** | **P3** | **P4** | **P5** | **P6** | **P7** | **P8** | **P9i** | **P9ii** | **P10** |
| --- | --- | --- | --- | --- | --- | --- | --- | --- | --- | --- | --- |
| **Age at onset (m)** | 1 | 2 | 4 | 5 | 7 | 5 | 12 | 8 | 5 | 12 | 10 |
| **Age at initial visit (m)** | 1 | 4 | 4 | 28 | 20 | 18 | 28 | 19 | 23 | 15 | 18 |
| **Ht SDS** | / | -2.30 | -2.61 | -4.44 | -2.98 | -2.10 | -3.89 | -3.74 | -4.61 | -3.63 | -3.84 |
| **Birth weight (g)** | 1895 | 3000 | 3000 | 3000 | 3000 | 3650 | 3300 | 2600 | 2980 | 2500 | 2700 |
| **FBG (mmol/L)** | 8.1 | 9.5 | 2.3 | 1.3 | 1.7 | 1.7 | 2.4 | 2.9 | 1.9 | 1.9 | 2.1 |
| **ALT (U/L)** | N | 39 | 32 | 555 | 125 | 38 | 53 | 146 | 131 | 69 | 42 |
| **AST (U/L)** | N | 180 | 100 | 1921 | 121 | 73 | 59 | 214 | 163 | 55 | 62 |
| **GGT (U/L)** | N | / | 170 | 338 | 169 | 55 | 36 | 122 | 137 | 46 | / |
| **TC (mmol/L)** | / | 2.25 | / | 7.30 | / | 5.78 | 4.05 | / | 7.16 | 6.12 | / |
| **TG (mmol/L)** | / | 4.31 | / | 3.16 | / | 3.79 | 3.39 | 6.17 | 2.17 | 2.92 | / |
| **UA SDS** | / | / | / | -3.5 | -3.5 | -3.5 | -3.1 | / | -3.1 | -3.6 | -3.3 |
| **Glycosuria** | +++ | ++++ | ++++ | +++ | +++ | +++ | +++ | ++++ | ++++ | +++ | ++++ |
| **Proteinuria** | - | - | + | ++ | - | ++ | + | - | + | ++ | + |
| **Ketonuria** | - | - | - | +- | +- | - | ++ | - | ++ | +++ | ++ |
| **pH** | 7.29 | 7.42 | 7.34 | 7.30 | 7.41 | 7.35 | 7.35 | 7.34 | 7.35 | 7.34 | 7.36 |
| **HCO_3_^-^ (mmol/L)** | 11.4 | 17.9 | 15.8 | 11.8 | 17.7 | 16.1 | 19.4 | 13.4 | 9.7 | 18.2 | 13.6 |
| **BE** | -10.6 | -7.0 | -7.8 | -12.6 | -6.9 | -8.3 | -6.2 | -10.8 | -11.7 | -12.6 | -11.8 |
| **Lactate (mmol/L)** | / | / | / | / | 2.9 | 2.6 | / | 2.8 | 1.8 | 0.8 | 2.1 |
| **AKP SDS** | N | 36.7 | 32.7 | 5.6 | 8.1 | 5.4 | 4.6 | 10.1 | 10.4 | 7.3 | 6.7 |
| **Phosphorus SDS** | / | -5.7 | -6.1 | -7.8 | -4.7 | -4.7 | -6.4 | -5.6 | -7.7 | -6.0 | -7.0 |
| **Calcium (mmol/L)** | / | 2.65 | 2.20 | 2.40 | 2.58 | 2.43 | 2.23 | 2.21 | 2.24 | 2.63 | 2.29 |
| **Hepatomegaly** | No | Yes | Yes | Yes | Yes | Yes | Yes | Yes | Yes | Yes | Yes |
| **Short stature** | No | Yes | Yes | Yes | Yes | Yes | Yes | Yes | Yes | Yes | Yes |
| **Hypophosphataemic rickets** | No | No | No | Yes | No | Yes | Yes | Yes | Yes | Yes | Yes |

m: month. FBG: fasting blood glucose; ALT: alanine aminotransferase; AST: aspartate transaminase; GGT: γ-glutamyl transferase; TC: total cholesterol; TG: triglyceride; UA SDS: uric acid standard deviation score; HCO_3_^-:^ plasma bicarbonate; BE: base excess; AKP SDS: alkaline phosphatase SDS. N: normal but lacking specific values; /: not available; No: the patient did not present with this symptom; Yes: the patient presented with this symptom.**Table S3.** Clinical and biochemical information of 11 patients with FBS at diagnosis

|  | **P1** | **P2** | **P3** | **P4** | **P5** | **P6** | **P7** | **P8** | **P9i** | **P9ii** | **P10** |
| --- | --- | --- | --- | --- | --- | --- | --- | --- | --- | --- | --- |
| **Age at diagnosis (y)** | 1.1 | 3.4 | 1.5 | 3.4 | 1.7 | 2.3 | 2.3 | 2.8 | 2.4 | 1.3 | 1.8 |
| **Ht SDS** | -1.93 | / | -5.85 | -4.49 | -4.27 | -2.35 | -3.89 | -4.63 | -4.82 | -3.63 | -4.84 |
| **PBG (mmol/L)** | 3.3 | 2.3 | 1.4 | 1.4 | 1.9 | 1.7 | 2.4 | 2.9 | 1.8 | 1.9 | 1.1 |
| **ALT (U/L)** | 47 | 529 | 61 | 127 | 125 | 38 | 53 | 146 | 210 | 69 | 60 |
| **AST (U/L)** | 99 | 866 | 152 | 427 | 121 | 73 | 59 | 214 | 270 | 55 | 283 |
| **GGT (U/L)** | 43 | / | 51 | 288 | 169 | 55 | 36 | 122 | 116 | 46 | 32 |
| **TC (mmol/L)** | 5.95 | 4.11 | 1.07 | 5.86 | 2.79 | 5.78 | 4.05 | 4.20 | 6.00 | 6.12 | 4.88 |
| **TG (mmol/L)** | 6.68 | 1.21 | 1.05 | 2.43 | 1.38 | 3.79 | 3.39 | 6.17 | 2.09 | 2.92 | 3.62 |
| **UA SDS** | -2.89 | -3.35 | -3.38 | -3.18 | -3.11 | -3.48 | -3.11 | -3.46 | -2.58 | -3.56 | -2.49 |
| **Glycosuria** | ++ | ++ | ++++ | +++ | +++ | +++ | +++ | ++++ | ++++ | +++ | ++++ |
| **Proteinuria** | - | ++ | - | ++ | - | ++ | + | - | ++ | ++ | ++ |
| **Ketonuria** | + | ++ | +++ | +++ | +- | - | ++ | - | ++ | +++ | - |
| **pH** | 7.42 | 7.31 | 7.29 | 7.33 | 7.41 | 7.35 | 7.35 | 7.34 | 7.29 | 7.34 | 7.23 |
| **HCO_3_^-^ (mmol/L)** | 16.2 | 13.1 | 19.8 | 12.6 | 17.7 | 16.1 | 19.4 | 13.4 | 12.1 | 14.6 | 11.3 |
| **BE** | -8.3 | -11.9 | -6.4 | -11.4 | -6.9 | -8.3 | -6.2 | -10.8 | -12.9 | -12.6 | -14.9 |
| **Lactate (mmol/L)** | 2.3 | / | 2.2 | 1.2 | 1.4 | 2.6 | 1.8 | 2.8 | 1.8 | 0.8 | 1.7 |
| **AKP SDS** | 17.2 | 3.9 | 4.9 | 9.2 | 8.1 | 5.4 | 4.6 | 10.1 | 10.8 | 7.3 | 10.0 |
| **Phosphorus SDS** | -8.3 | -6.7 | -7.5 | -9.2 | -4.7 | -4.7 | -6.4 | -5.6 | -7.9 | -6.0 | -4.9 |
| **Calcium (mmol/L)** | 2.35 | 2.27 | 2.22 | 2.26 | 2.58 | 2.43 | 2.23 | 2.21 | 2.26 | 2.63 | 2.26 |

y: year. PBG: pre-prandial blood glucose; ALT: alanine aminotransferase; AST: aspartate transaminase; GGT: γ-glutamyl transferase; TC: total cholesterol; TG: triglyceride; UA SDS: uric acid standard deviation score; HCO_3_^-^: plasma bicarbonate; BE: base excess; AKP SDS: alkaline phosphatase SDS. /: not available

**Table S4.** Clinical and biochemical information of 11 patients with FBS at the last follow-up

|  | **P1** | **P2** | **P3** | **P4** | **P5** | **P6** | **P7** | **P8** | **P9i** | **P9ii** | **P10** |
| --- | --- | --- | --- | --- | --- | --- | --- | --- | --- | --- | --- |
| **Age at the last follow-up(y)** | 16.6 | / | / | 15.6 | 10.7 | 9.5 | 8.9 | 5.3 | 4.4 | 3.1 | 5.7 |
| **Ht SDS** | -9.14 | / | / | -4.09 | -3.67 | -2.37 | -2.68 | -3.52 | -4.54 | -3.26 | -3.68 |
| **PBG (mmol/L)** | 3.8 | / | / | 3.7 | 4.3 | 5.4 | 4.6 | 5.6 | 4.7 | 4.3 | 3.7 |
| **ALT (U/L)** | 33 | / | / | 49 | 21 | 34 | 24 | 28 | 27 | 22 | 36 |
| **AST (U/L)** | 23 | / | / | 106 | 34 | 122 | 28 | 66 | 32 | 33 | 51 |
| **GGT (U/L)** | 316 | / | / | 288 | 43 | 44 | 34 | 49 | 17 | 36 | 21 |
| **TC (mmol/L)** | 6.51 | / | / | 5.95 | 4.58 | 4.10 | 4.36 | 5.18 | 4.82 | 6.90 | 4.46 |
| **TG (mmol/L)** | 2.31 | / | / | 1.50 | 1.14 | 1.71 | 0.66 | 1.36 | 1.27 | 1.36 | 1.25 |
| **UA SDS** | -3.9 | / | / | -3.7 | -3.6 | -3.5 | -3.4 | -2.4 | -2.9 | -3.2 | -2.5 |
| **Glycosuria** | ++++ | / | / | ++++ | ++++ | ++++ | ++++ | ++++ | ++++ | +++ | ++++ |
| **Proteinuria** | + | / | / | ++ | + | +- | + | - | + | + | + |
| **Ketonuria** | +++ | / | / | ++ | +++ | - | - | + | - | - | + |
| **pH** | 7.20 | / | / | 7.28 | 7.39 | 7.35 | 7.40 | 7.33 | 7.28 | 7.28 | 7.35 |
| **HCO_3_^-^ (mmol/L)** | 12.9 | / | / | 15.0 | 22.8 | 23.7 | 23.5 | 21.7 | 14.3 | 15.1 | 21.4 |
| **BE** | -16.1 | / | / | -10.5 | -2.7 | -1.8 | -1.9 | -4.1 | -13.5 | -11.8 | -3.8 |
| **Lactate (mmol/L)** | 1.1 | / | / | 1.1 | 0.7 | 1.6 | 1.8 | 1.4 | 1.7 | 2.8 | 1.6 |
| **AKP SDS** | 18.7 | / | / | 3.6 | 2.2 | 0.9 | 2.7 | 4.2 | 7.7 | 1.1 | 4.9 |
| **Phosphorus SDS** | -6.3 | / | / | -7.1 | -5.8 | -5.6 | -6.6 | -3.7 | -5.2 | -5.4 | -4.8 |
| **Calcium (mmol/L)** | 2.19 | / | / | 2.25 | 2.39 | 2.40 | 2.50 | 2.49 | 2.63 | 2.68 | 2.32 |

y: year. PBG: pre-prandial blood glucose; ALT: alanine aminotransferase; AST: aspartate transaminase; GGT: γ-glutamyl transferase; TC: total cholesterol; TG: triglyceride; UA SDS: uric acid standard deviation score; HCO_3_^-:^ plasma bicarbonate; BE: base excess; AKP SDS: alkaline phosphatase SDS. /: not available

**Table S5.** *SLC2A2* variants in 11 patients with FBS

|  |  |  | **Allele 1 (paternal)** | | | | **Allele 2 (maternal)** | | | |
| --- | --- | --- | --- | --- | --- | --- | --- | --- | --- | --- |
| **Patient ID** | **Paternal origin** | **Maternal origin** | **Nucleotide alteration (NM_000340.1)** | **Amino acid alteration** | **Classification** | **ref.** | **Nucleotide alteration (NM_000340.1)** | **Amino acid alteration** | **Classification** | **ref.** |
| P1 | Jiangsu | Jiangsu | c.609T>A | p.S203R | LP | ^1^ | c.609T>A | p.S203R | LP | ^1^ |
| P2 | Jiangsu | Jiangsu | c.1313dup | p.A439SfsX45 | P | ^2^ | c.379_380insTC | p.A127VfsX4 | P | ^2^ |
| P3 | Guangdong | Guangdong | c.1217T>G* | p.L406R | LP | this study | c.1217T>G* | p.L406R | LP | this study |
| P4 | Guangxi | Guangxi | c.255_256del | p.E85DfsX93 | P | ^3^ | c.496+2T>G | / | P | this study |
| P5 | Sichuan | Sichuan | c.416del | p.A139VfsX3 | P | ^4^ | c.485G>T | p.G162V | LP | this study |
| P6 | Jiangxi | Jiangxi | c.1217T>G | p.L406R | LP | this study | c.1217T>G | p.L406R | LP | this study |
| P7 | Hunan | Hunan | c.566G>A | p.G189D | LP | ^5^ | c.566G>A | p.G189D | LP | ^5^ |
| P8 | Henan | Henan | E10-11del^ | / | LP | this study | c.1106_1109del | p.F369X | P | this study |
| P9i | Fujian | Fujian | c.553_554del | p.R185GfsX18 | P | this study | c.553_554del | p.R185GfsX18 | P | this study |
| P9ii | Fujian | Fujian | c.553_554del | p.R185GfsX18 | P | this study | c.553_554del | p.R185GfsX18 | P | this study |
| P10 | Jiangxi | Jiangxi | c.1093C>T | p.R365X | P | ^6^ | c.371+2T>C# | / | P | ^7^ |

P: pathogenic; LP: likely pathogenic; /: unknown amino acid alteration; *: variant with unknown origin; #: variant de novo; ^: the gross deletion has been verified by quantitative PCR.

1. Sansbury, F. H.; Flanagan, S. E.; Houghton, J. A.; Shuixian Shen, F. L.; Al-Senani, A. M.; Habeb, A. M.; Abdullah, M.; Kariminejad, A.; Ellard, S.; Hattersley, A. T., SLC2A2 mutations can cause neonatal diabetes, suggesting GLUT2 may have a role in human insulin secretion. *Diabetologia* **2012,** *55* (9), 2381-5.

2. YE., D.; QQ., L.; X., S.; W., G.; SN, N., Case report of Fanconi-Bickel syndrome and its clinical feature. *Annual Congress of Pediatric Society* **2012**, 408.

3. Al-Haggar, M.; Sakamoto, O.; Shaltout, A.; El-Hawary, A.; Wahba, Y.; Abdel-Hadi, D., Fanconi Bickel Syndrome: Novel Mutations in GLUT 2 Gene Causing a Distinguished Form of Renal Tubular Acidosis in Two Unrelated Egyptian Families. *Case Rep Nephrol* **2011,** *2011*, 754369.

4. Xiong, L.-J.; Jiang, M.-L.; Du, L.-N.; Yuan, L.; Xie, X.-L., Fanconi-Bickel syndrome in an infant with cytomegalovirus infection: A case report and review of the literature. *World J Clin Cases* **2020,** *8* (21), 5467-5473.

5. Sharari, S.; Abou-Alloul, M.; Hussain, K.; Ahmad Khan, F., Fanconi-Bickel Syndrome: A Review of the Mechanisms That Lead to Dysglycaemia. *International journal of molecular sciences* **2020,** *21* (17).

6. Sharari, S.; Kabeer, B.; Mohammed, I.; Haris, B.; Pavlovski, I.; Hawari, I.; Bhat, A. A.; Toufiq, M.; Tomei, S.; Mathew, R.; Syed, N.; Nisar, S.; Maacha, S.; Grivel, J. C.; Chaussabel, D.; Ericsson, J.; Hussain, K., Understanding the Role of GLUT2 in Dysglycemia Associated with Fanconi-Bickel Syndrome. *Biomedicines* **2022,** *10* (9).

7. Santer, R.; Groth, S.; Kinner, M.; Dombrowski, A.; Berry, G. T.; Brodehl, J.; Leonard, J. V.; Moses, S.; Norgren, S.; Skovby, F.; Schneppenheim, R.; Steinmann, B.; Schaub, J., The mutation spectrum of the facilitative glucose transporter gene SLC2A2 (GLUT2) in patients with Fanconi-Bickel syndrome. *Hum Genet* **2002,** *110* (1), 21-9.
