# Supplementary material for: Clinical, genetic profile and therapy evaluation of 11 Chinese pediatric patients with Fanconi-Bickel syndrome
Source: Orphanet J Rare Dis. 2024 Feb 16;19:75. doi: 10.1186/s13023-024-03070-8 (PMC10874070; doi:10.1186/s13023-024-03070-8)
Supplement: Supplementary file 2 — Additional file 2. The detailed genetic test procedures. [file 13023_2024_3070_MOESM2_ESM.docx]

The genetic test procedures were as follows:

(i) Sanger sequencing: the entire coding regions and splice sites of the *SLC2A2* gene were amplified by PCR using appropriate primers; the primer sequences are available upon request. Amplified fragments were sequenced using a 96-capillary 3730xl system (ABI, Foster City, California, United States). (ii) ES: ES was performed using the capture kit of the xGen Exome Research Panel (Integrated DNA Technologies, Coralville, IO, USA), as previously reported ^1^. Variants at a frequency over 1% in 1000 Genomes Project, Genome Aggregation Database (GnomAD), and Exome Variant Server (EVS) or at a frequency over 5% in a local database (containing approximately 6000 exomes) were excluded from the list of candidate variants. XHMM was applied to call copy number variations (CNVs) ^2^. The H^3^M^2^ algorithm was used to index patient ES data for detecting regions of homozygosity (ROH). Variants were finally classified following the guidelines of the American College of Medical Genetics and Genomics (ACMG) ^3^. Sanger sequencing was further performed to confirm the variants detected by ES and identify their parental origins. A heterogenous deletion of exons 10-11 found in P8 was confirmed by quantitative polymerase chain reaction (qPCR). The primer sequences and PCR conditions used are available upon request.

Reference:

1. Xia, Y.; Duan, Y.; Zheng, W.; Liang, L.; Zhang, H.; Luo, X.; Gu, X.; Sun, Y.; Xiao, B.; Qiu, W., Clinical, genetic profile and therapy evaluation of 55 children and 5 adults with sitosterolemia. *J Clin Lipidol* **2022,** *16* (1), 40-51.

2. Fromer, M.; Moran, J. L.; Chambert, K.; Banks, E.; Bergen, S. E.; Ruderfer, D. M.; Handsaker, R. E.; McCarroll, S. A.; O'Donovan, M. C.; Owen, M. J.; Kirov, G.; Sullivan, P. F.; Hultman, C. M.; Sklar, P.; Purcell, S. M., Discovery and statistical genotyping of copy-number variation from whole-exome sequencing depth. *Am J Hum Genet* **2012,** *91* (4), 597-607.

3. Richards, S.; Aziz, N.; Bale, S.; Bick, D.; Das, S.; Gastier-Foster, J.; Grody, W. W.; Hegde, M.; Lyon, E.; Spector, E.; Voelkerding, K.; Rehm, H. L., Standards and guidelines for the interpretation of sequence variants: a joint consensus recommendation of the American College of Medical Genetics and Genomics and the Association for Molecular Pathology. *Genet Med* **2015,** *17* (5), 405-424.
